# Assessment of Female Sexual Function Following Transobturator Midurethral Sling for Stress Urinary Incontinence 12 and 36 Months After Surgical Treatment in Postmenopausal Women

**DOI:** 10.3390/jcm14092965

**Published:** 2025-04-25

**Authors:** Gabriela Kołodyńska, Maciej Zalewski, Joanna Lewandowska, Anna Mucha, Aleksandra Piątek, Waldemar Andrzejewski

**Affiliations:** 1 Department of Physiotherapy, Wroclaw University of Health and Sport Sciences, 51-612 Wrocław, Polandwaldemar.andrzejewski@awf.wroc.pl (W.A.); 2 Department of Gynaecology and Obstetrics, Faculty of Health Sciences, Medical University of Wrocław, 50-367 Wrocław, Poland; zalewski@interia.pl; 3 Independent Public Health Care Center of the Ministry of the Interior and Administration in Wrocław, Department of Gynaecology, 50-233 Wrocław, Poland; 4 Department of Genetics, Wrocław University of Environmental and Life Sciences, 50-375 Wrocław, Poland; anna.mucha@upwr.edu.pl

**Keywords:** urinary incontinence, sling procedure, sexual function, menopause

## Abstract

**Background/Objectives**: Stress urinary incontinence (SUI) is a common condition affecting sexual function, exercise activities, and quality of life, accompanied by psychological distress. The treatment of SUI includes conservative and surgical treatment which comprises tensions-free vaginal tapes in the form of retropubic (TVT) and transobturator tape (TOT or TVT—O). The TVT procedure is considered the gold standard, but the TOT procedure is a safe alternative due to a lower rate of bladder and bowel complications. The aim of this study is to evaluate the long-term effects of the surgical treatment of the TOT procedure after 36 months of follow-up on the sexuality of women with SUI. **Methods**: In the long-term evaluation, 3 years after surgery, 45 women with medical records sufficient for analysis participated in the project. The international standardized Female Sexual Function Index (FSFI) self-administered questionnaire was completed three times: before the surgery, 12 months after surgery, and 3 years after surgery. **Results:** Domain scores for arousal, lubrication, orgasm, and total score were lower in the follow-up group than before and after the surgery. The pain domain was the highest in the after-surgery group, slightly lower in the before-surgery group, and the lowest in the follow-up group. All the abovementioned differences were statistically significant. **Conclusions:** Three years after the surgical treatment of SUI, the results of our study patients deteriorated, especially in arousal, lubrication, and orgasm assessments, compared to outcomes 12 months after the procedure. In addition, the total FSFI score was lower 36 months after the surgery than the year before the surgery and lower than before the procedure. Also, pain during intercourse was more frequently reported, as opposed to the result year after the surgery, which was lower than before the operation.

## 1. Introduction

Urinary incontinence (UI), defined as the involuntary leakage of urine, is a common condition in the general female population. The prevalence of UI estimates between 25% and 45% in women and 5% and 45% in men internationally [1], approximately 424 million people worldwide—303 million women and 121 million men [2]. The prevalence has been found to increase with age [3]. Chronic or strong intraabdominal pressure lifting actions are associated with a risk of pelvic floor and urethral dysfunction, e.g., obesity, lifting heavy objects, exercise, but also vaginal delivery or trauma [4] In Poland, the prevalence of UI estimates between 21.3 and 36.6,% of women. And yet, many cases of UI go untreated [5]. According to the World Health Organization (WHO), the UI is one of the priorities in the health field as it may lead to physical, psychological, and social consequences [4].

Urinary incontinence in women occurs most often as stress, urgency, and mixed incontinence. The most common type is stress urinary incontinence (SUI), which causes the involuntary leakage of urine due to increased intraabdominal pressure (coughing, sneezing, laughing, jumping, effort, or exertion) [5]. SUI symptoms affect sexual function, exercise activities, and quality of life, and are accompanied by psychological distress, social isolation, and depression symptoms, especially in older women [6]. Physical complications include sleep disturbances and a decrease in sleep quality, ulcers, infections of the urinary tract, and risk of falls and fractures, leading to increased health care costs, overall morbidity, and mortality [4,7,8].

A reduction in the quality of life is associated with a loss of bladder control, affecting daily and social activities, physical exercise, and sexual function [9].

Sexual health is an important part of the overall quality of life, affecting emotional well-being, self-worth, and cognitive function [10]. Sexual difficulties and dysfunctions are common in women with pelvic floor disorders [11] and are reported in 46% of patients with UI [12]. Discomfort caused by urine leakage may be a source of embarrassment in the sexual sphere. Women experiencing UI may avoid intimate sexual relations depending on the kind of UI, severity of the symptoms, and personal attitude [6]. Decreased libido and lower self-esteem in women reporting UI might be due to menopausal vaginal dryness and dyspareunia, defined as genital pain that can be experienced before, during or after intercourse [10]. Nonetheless, clinical observations suggest that urgency and mixed UI are more detrimental and have a greater negative impact on sexual function than SUI [13,14]. The reason for the higher anxiety level in urge incontinence is the lack of predictability of a leakage and thus the withdrawal from intimate relationships.

The treatment of SUI includes conservative and surgical treatment. Conservative UI treatment involves lifestyle strategies such as adequate fluid intake, avoiding alcohol consumption and reducing caffeine [3,8], tampons or pessaries and vaginal estrogen therapy [15]. The International Continence Society (ICS) recommends pelvic floor muscle training as a first-choice treatment [16]. Other conservative treatments include physical therapy to improve the strength and reeducation of pelvic floor muscles, pharmacotherapy, and behavioral therapy.

Surgical methods should be implemented when a conservative approach is not effective. The surgical treatment comprises tensions-free vaginal tapes (TVT), i.e., retropubic and transobturator tape (TOT or TVT—O). The TVT procedure is considered the gold standard, but the TOT procedure is a safe alternative due to a lower rate of bladder and bowel complications compared to [17,18,19]. However, in spite of being a viable treatment option, meshes, including mid-urethral slings, are not advised as a first-line treatment in several countries. Alternative treatment includes Burch colposuspension, urethral bulking agents, and autologous fascial slings [20].

The aim of this study is to evaluate the long-term effects of the surgical treatment of the transobturator tape (TOT) procedure after 36 months of follow-up on the sexuality of women with stress urinary incontinence (SUI).

## 2. Materials and Methods

This study initially involved 50 patients from the Gynaecology Department of the Hospital of the Ministry of Internal Affairs and Administration in Wroclaw, who were diagnosed with stress urinary incontinence and underwent treatment using the TOT method.

All patients were followed up for at least 3 years postoperatively [21].

This study was conducted according to the ethical standards laid down in the 1964 Declaration of Helsinki and its later amendments. The research was approved by the Bioethics Committee of the Medical University in Wroclaw with a number KB 346/2024. Patients signed informed consent to participate in this study.

The inclusion criteria for this study were a diagnosis of stress urinary incontinence and undergoing TOT surgical treatment at the Gynecology Department of the Hospital of the Ministry of Internal Affairs and Administration 3 years earlier, and participation in the assessment 12 months after surgery. Patients were excluded if they did not follow up or if they declined to participate. Only patients who met the criteria of postmenopausal status were included in this study. Postmenopausal status was determined based on the patient’s history (absence of menstruation for at least 12 months) and, if needed, confirmed by hormonal testing (FSH and estradiol levels).

Of 50 patients, 3 years after surgery, only 45 had sufficient medical records for the analysis. The remaining 5 women did not participate in the follow-up study due to death, illness, change of place of residence, and reluctance to fill out the questionnaire again.

The international standardized Female Sexual Function Index (FSFI) self-administered questionnaire was filled out three times: before the surgery, 12 months after surgery, and 3 years after surgery.

All the patients were followed up in an outpatient clinic every 12 months and were using local estrogen (cream or globules) two times a week. Correct localization of the tape was confirmed by ultrasound examination (positioned at the midpoint of the urethra, 3–5 mm from the urethra) during every examination.

The FSFI is a 19-item measure providing scores on overall sexual function, and its primary components are sexual desire, arousal, orgasm, pain, and satisfaction. The questionnaire concerns the patient’s sexual contacts in the previous 4 weeks. Pain domain results are scored using a descending scale, which means that higher results are associated with better functioning.

All patients qualified for the project had a TOT implementation procedure outside-in technique using the ABBIS CYRENE sling. A detailed surgical protocol was described in our previous publication [21]. In all cases, the procedure was successful concerning UI symptoms. Correct localization of the tape was confirmed by ultrasound examination.

All statistical analysis was performed in R 4.3.1 [22]. Statistical inference was made at the level of significance α = 0.05.

The reliability of the questionnaire was verified on the basis of Cronbach’s alpha in the ltm [23] package. This part of the analysis was performed for the entire questionnaire and all its domains separately. All Cronbach’s alpha values were tested for statistical significance. Then, it was verified whether the differences in the value of this parameter between domains and between groups (before surgery, after surgery, and follow-up) were statistically significant. For this purpose, a comparison of confidence intervals with Bonferroni correction [24] was used.

The answers to individual questions obtained from the surveyed patients were transformed according to the Likert scale. Then, the answer points for questions corresponding to individual domains were summed up. The resulting sums were unitarized with zero minimum, creating domain scores.

The compliance of the domain scores distribution with the normal distribution was verified using the Shapiro–Wilk test. Due to the lack of such compliance, the statistical significance of the impact of the surgical procedure on the values of particular domains and the total score was verified based on the Friedman test (non-parametric repeated measures ANOVA). The size of the effect was also determined [25]. The necessary *post hoc* analysis was performed using the paired Wilcoxon test with Bonferroni correction. This part of the analysis was performed in the rstatix [26] package.

The analysis of the relationship between individual domains was performed using Spearman’s rank correlation analysis. The statistical significance of each determined correlation was verified, taking into account the Bonferroni correction. Correlation analysis was performed for the entire dataset and then for three time points separately (before surgery, after surgery, and follow-up). Corresponding correlation coefficients were compared based on Bonferroni-corrected confidence intervals [24]. This part of the analysis was performed in the psych [27] package.

Principal components analysis (PCA) was performed to verify the differentiation between the analyzed groups (before surgery, after surgery, follow-up) and to find out which domains differentiated the analyzed groups the most. This part of the analysis was performed in the ade4 [28] and factoextra [29] packages.

## 3. Results

In the long-term evaluation, 3 years after surgery, 45 women with medical records sufficient for analysis participated in the project. The mean age of the patients was 59.4 years, and the range was 48–63 years. The group characteristics are shown in Table 1.

During the 36-month follow-up, none of the 45 patients reported recurrence of stress urinary incontinence symptoms, nor de novo urge incontinence. This was confirmed through clinical examination and patient self-reporting.

**Figure 1:**
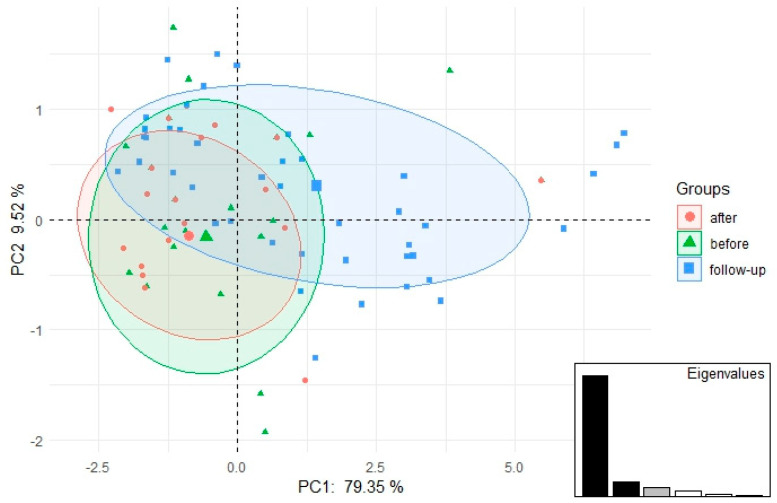
Scatterplot of PCA results (FSFI questionnaire domain scores) for women before surgery, after surgery, and follow-up.

The distribution of responses to individual domains of the FSFI questionnaire and the correlations before, after, and follow-up are presented in Table 2, Table 3, Table 4, Table 5 and Table 6 and Figure 1.

The reliability of the questionnaire was statistically significantly higher in the case of the arousal domain for the follow-up group than for the same domain for the other two groups. No other significant differences in Cronbach’s alpha values were detected (Table 2).

Data are presented as median (range) and mean (standard deviation).

Given *p*-values are for the Friedman test (non-parametric analysis of variance for dependent samples).

Statistically significant differences between groups were marked by different letters (paired Wilcoxon test for dependent samples with Bonferroni correction, *p*-value < 0.05).

Domain scores for arousal, lubrication, orgasm, and total score were lower in the follow-up group than in the other two groups. The pain domain was the highest in the after-surgery group, slightly lower in the before-surgery group, and the lowest in the follow-up group. All the abovementioned differences were statistically significant (Table 3).

The relationship between desire and arousal domains was statistically significantly higher in the follow-up group than in the after-surgery group. Analogous differences have been demonstrated for the relationship between arousal and orgasm domains and satisfaction and pain domains. The correlation of desire and lubrication domains, desire and orgasm domains, desire and satisfaction domains, arousal and satisfaction domains, lubrication and orgasm domains, and lubrication and satisfaction domains was higher in the follow-up group than in the two remaining groups. The arousal and lubrication domains were strongly correlated in the follow-up group than before surgery. For the after-surgery group, a higher correlation was determined for the lubrication and pain domains than before surgery. The relationship of orgasm and pain domains was higher in the after-surgery group than in the two remaining groups (Table 4 and Table 5).

**Figure 2:**
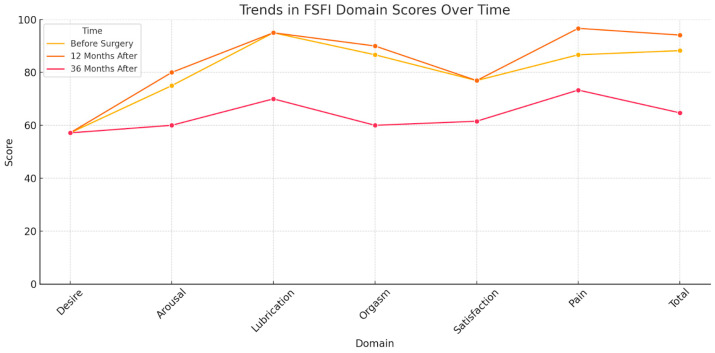
Changes in FSFI domain scores at three time points: before surgery, 12 months after the surgery, and 36 months after the surgery. Score results are presented in percents (100% is the maximum score of 36 points).

The first two main components were taken into account because they explained as much as 88.87% of the variability (Table 6). All domain scores were highly negatively correlated with the first principal component (Table 6), which means that the shift of the ellipse to the right means a decrease in domain scores, which in turn means that the lowest domain scores were recorded in the follow-up group (Figure 2). Changes of the FSFI score over time are presented in Figure 2.

## 4. Discussion

Urinary incontinence is a common condition that affects women of all ages. UI has a significant impact on sexual health due to its comorbidity with other pelvic floor dysfunctions, such as pelvic organ prolapse [30]. Additionally, aging, menopausal status, and the presence of comorbidities negatively influence sexual function [31].

Although this study aimed to include only postmenopausal women, there might be a difference in the severity of genitourinary symptoms in patients depending on the time since last menstruation. This factor might partly explain discrepancies with other studies.

Bekker et al. observed significantly lower overall sexual function, lower frequency of intercourse, more problems with communication, and the tendency to avoid intercourse in women with UI [32]. The majority of patients with UI report lower desire and satisfaction, anxiety of odor and coital UI, as well as orgasmic problems [33].

Not only was women’s lower overall sexual function, higher avoidance behavior, and lower frequency of sexual intercourse and satisfaction observed. Also, female stress urinary incontinence also negatively affected their partner’s sexual function [34].

Several studies reported positive and negative influences of surgical treatment in this field. The effectiveness of surgical treatment is focused mainly on the treatment of UI, not giving a deeper insight into patients’ sexual function. It is believed that the treatment of UI improves sexual function due to cessation of incontinence during intercourse, but, at the same time, may worsen sexual function due to dyspareunia.

This hypothesis coincides with our study, where 47 women were examined, showing no significant differences in the Female Sexual Function Index Questionnaire (FSFI) domain scores before and 6 months after mid-urethral sling procedure [35].

Similarly, Jang et al. reported no significant change after 36-month follow-up after surgery in overall sexual function in all 47 women who underwent the mid-urethral procedure for SUI (transobturator route and retropubic route). Less pain during post-surgery intercourse was observed in patients who underwent the TOT procedure [36].

In contrast, there are many studies suggesting a positive impact of mid-urethral surgeries on sexual function.

Our long-term findings generally do not align with existing studies, including the meta-analysis of 22 studies assessing the influence of mid-urethral sling surgery on UI, which shows the positive impact of this procedure on women’s sexual health. There was a significant improvement in Pelvic Organ Prolapse-Urinary Incontinence Sexual Function Questionnaire (PISQ-12) scores and FSFI total score, as well as in FSFI sub-scores (desire, arousal, orgasm, lubrication, satisfaction, and pain during intercourse) in all analyzed studies. Scores were significantly higher than preoperative scores during 6-month, 12-month, and 24-month follow-up measurement points [37].

A great majority of studies assessing sexual function in short-term follow-up (up to 12 months) report notable improvement [21,38,39,40,41,42,43].

Soon after the TOT procedure, Kamalak et al. evaluated changes in the sexual function of women with SUI. Significant improvement in FSFI scores was reported, and a positive effect was observed in sexual desire, arousal, lubrication, orgasm, and satisfaction 3 months after surgery. Moreover, there was a significant decrease in pain during and after intercourse [38].

In our previous study, premenopausal and postmenopausal women who underwent TOT surgery were evaluated at two measurement points (before surgery and 6 months after). There was a significant decrease in pain and discomfort during intercourse and improvement in lubrication [21]. After a similar time of follow-up, significant improvement in satisfaction and pain, but not on a significant level, in other sub-domains of the FSFI score, was reported in a Turkish study [39].

Also, half a year after the TVT procedure, 80 women diagnosed with SUI were examined. According to the Patient’s Global Impression of Improvement Scale (PGI-I), there was significant improvement in 86% of women. Also, FSFI total score and sub-scores (sexual desire, lubrication, arousal) were higher after surgery [40].

Positive changes after a year were reported by Mengerink et al. Mid-urethral sling (TVT or TOT) surgery resulted in overall positive influence on sexual function, including significant decrease in coital incontinence and increase in overall sexual activity [41].

In another study concerning TVT and TOT procedure, there was an improvement in sexual function in 86% of patients who underwent either of the procedures, whereas in all women, no worsening of sexual activity or function was observed after surgery [42]. Similarly, a significant decrease in coital incontinence and improvement in FSFI total score and sub-score (desire) were demonstrated in the TOT group [43].

Even in a period of 24 months, there was reported improvement in sexual health according to FSFI scores of women who underwent surgical SUI treatment (TOT, Perigee, or MiniArc). All women reported a decrease in UI symptoms after surgery, as well as improvement in desire, arousal, and orgasm frequency [44].

However, some evidence appears to support our conclusion that even though short-term outcomes stand for sexual function improvement, results after more than one year demonstrate continuous deterioration in all or almost all domains.

A prospective cohort study by Kender Erturk et al. shows an impact of the transobturator tape procedure on SUI and female sexual function. The sexual function of 70 women was assessed at three measurement points: before surgery, 6 months, and 24 months after. The total score of FSFI before surgery was lower in women with SUI compared to controls. There was a slight increase in sexual functions in the other two measurement points; at the same time, the FSFI total score in the control group significantly decreased [45].

A growing body of analyses reveals that the improvement of sexual functioning relates to the cure of coital incontinence, and the major reason for the worsening is dyspareunia [46]).

Short-term improvement and following gradual deterioration might have been associated with local estrogen treatment, which is recommended for every patient after the procedure. Lower compliance with recommendations is the reason of recurrence of genitourinary symptoms, resulting in vaginal dryness, pruritus and dyspareunia. Correlation between local estrogen therapy and the improvement of sexual function was proved [47].

Transobturator tape surgery is one of the most performed surgeries for SUI owing to its relatively lower post-surgery complications. Moving on to reflect on change in reported pain, it is worth emphasizing that almost 40% of patients with stress UI report mild pelvic girdle pain. After the procedure of inside-out TOT implementation, 12% of all patients reported pain localized mainly in the lateral and medial parts of the leg, groin, and low back. Pain resolved in more than 99% of patients by the sixth postoperative week [48].

When compared to TVT, the TOT method was associated with more frequent (16% vs. 1.5%) and longer-lasting (average 5–10 days vs. 2 weeks) groin pain [49].

Long-term outcomes indicate that 10% of women after the procedure have occasional pelvic pain and 2% have perineal pain. Even a few years after the procedure, among sexually active individuals, 6% reported pain during intercourse and 4–9% dyspareunia [50,51].

Although in anatomical studies no pudendal nerve branches were injured after the procedure [52], more recent studies show that alteration in anterior wall anatomy resulted in a significant decrease of cold, warm and vibratory sensation at the clitoral region and decrease feeling to warm stimuli in the anterior vaginal wall [53]. Also, it might affect the densely innervated G-spot area [54]

Mechanisms which may play role in the worsening of sexual function after sling procedures comprise dyspareunia, sensation alterations, decreased/increased lubrication and anorgasmia due to innervation changes, but also voiding dysfunction, vaginal narrowing, or erosions [46].

Importantly, taking into consideration sexual comfort, implanted TVT has a V-shaped course, compared to more horizontally placed TOT, which might be important for the risk of erosion, dyspareunia, and sling “feeling” by the partner. Thus, TVT is considered a recommended sling for sexually active patients.

Major limitations of this study are a lack of a control group and not taking into account psychosocial factors of pivotal importance to patients’ well-being, as well as different comorbidities that might impact sexual function in perimenopausal patients.

It should be noted that a temporary improvement in sexual function reflects the resolution of SUI symptoms. Even though the procedure is considered effective in a long-term study [55], possible recurrence of SUI symptoms might contribute to the progressive decline in sexual function in prolonged observations.

Also, it is worth considering patients’ comorbidities, which may strongly affect their intimate life. Diabetes may cause vascular damage and through endothelial dysfunction hinder vaginal and clitoral blood flow, resulting in dryness [56]. A similar effect is recognized in patients with hypertension due to arterial wall stiffness. Longer duration of the disease and uncontrolled hypertension are associated with lower FSFI scores, but treatment with beta-blockers and diuretics may also negatively affect sexual function [57].

Also, neurological diseases and diabetic neuropathies are contributing to impaired flow regulation and decreased genital sensation hormonal imbalances. Hormonal imbalances caused by hyperglycemia cause reduced libido [56].

To summarize, the preliminary results regarding the impact of surgical treatment on women’s sexual life seem promising. However, it is necessary to establish precise criteria and diagnostic tools that require extensive research to fully respond to the growing needs of contemporary women with stress urinary incontinence, also in terms of sexuality.

These studies were primarily aimed at emphasizing the importance of comprehensive care for patients with stress urinary incontinence. The patient’s mental state should be assessed before and after surgery, but also several years after it. These studies indicate that much attention should be paid to sexual issues.

## 5. Conclusions

1. Stress urinary incontinence can significantly affect the sexual life of women.
2. Three years after the surgical treatment of stress urinary incontinence, the results of this study’s patients deteriorated, especially in arousal, lubrication, and orgasm assessments, compared to outcomes 12 months after the procedure. In addition, the total FSFI score was lower 36 months after the surgery than the year before the surgery and lower than before the procedure. Also, pain during intercourse was more frequently reported, as opposed to the result year after the surgery, which was lower than before the operation.
3. The obtained results may indicate that TOT surgical treatment may not improve sexuality in the long term. However, this topic should be assessed in more detail, and a larger number of patients should be included in this study. Also, other variables that may affect the results should be considered, such as advancing age, comorbidities, post-menopausal symptoms, and change of working or relationship status.

## Figures and Tables

**Table 1:** Characteristics of the studied group.

Characteristics	Mean ± SD
Age (years)	59.4 ± 4.2
Weight (kg)	70.2 ± 10.8
Body height (cm)	162.1 ± 5.9
BMI (kg/m^2^)	26.7 ± 4.5
Underweight, *n* (%)	1 (2.2%)
Normal (healthy weight), *n* (%)	17 (37.8%)
Overweight, *n* (%)	18 (40.0%)
Obesity grade 1, *n* (%)	7 (15.6%)
Obesity grade 2, *n* (%)	2 (4.4%)

**Table 2:** Cronbach’s alpha for the FSFI questionnaire for the women before surgery, after surgery, and follow-up.

	Full Dataset	Before Surgery	After Surgery	Follow-Up
**FSFI total**	0.902	0.855	0.887	0.911
**desire**	0.924	0.925	0.917	0.941
**arousal**	0.912	0.851 ^a^	0.710 ^a^	0.972 ^b^
**lubrication**	0.948	0.956	0.847	0.960
**orgasm**	0.919	0.891	0.878	0.919
**satisfaction**	0.919	0.939	0.899	0.897
**pain**	0.904	0.872	0.863	0.909

Cronbach’s alphas differing statistically significantly between groups before surgery, after surgery, and follow-up were marked by different letters (α = 0.05). Cronbach’s alphas corresponding to particular domains of the FSFI questionnaire were not statistically significantly different (α = 0.05).

**Table 3:** FSFI questionnaire domain scores for the women before surgery, after surgery, and follow-up (n = 45).

	Before Surgery	After Surgery	Follow-Up	*p*-Value	Effect Size
**desire**	57.14 (0–100) 52.06 (24.40)	57.14 (0–100) 55.24 (24.29)	57.14 (0–100) 45.40 (34.23)	0.446	0.0179 (small)
**arousal**	75.00 ^a^ (20–95) 72.78 (18.26)	80.00 ^a^ (20–95) 76.44 (14.09)	60.00 ^b^ (0–100) 53.22 (29.33)	0.00308	0.129 (small)
**lubrication**	95.00 ^a^ (0–100) 87.22 (20.69)	95.00 ^a^ (20–100) 87.11 (16.70)	70.00 ^b^ (0–100) 59.89 (30.87)	3.6 × 10^−7^	0.330 (moderate)
**orgasm**	86.67 ^a^ (0–100) 80.59 (18.02)	90.00 ^a^ (20–100) 85.56 (13.74)	60.00 ^b^ (0–93.33) 52.15 (28.01)	9.81 × 10^−11^	0.512 (large)
**satisfaction**	76.92 (7.69–100) 75.90 (19.86)	76.92 (7.69–100) 78.97 (16.51)	61.54 (0–100) 59.15 (26.89)	0.000821	0.158 (small)
**pain**	86.67 ^b^ (20–100) 85.63 (15.17)	96.67 ^a^ (20–100) 89.41 (15.62)	73.33 ^c^ (0–100) 66.07 (30.25)	5.49 × 10^−5^	0.218 (small)
**TOTAL**	88.24 ^a^ (17.65–97.65) 84.39 (14.29)	94.12 ^a^ (17.65–100) 86.69 (15.36)	64.71 ^b^ (0–98.82) 59.58 (28.26)	8.88 × 10^−7^	0.310 (moderate)

Data are presented as median (range) and mean (standard deviation). Given *p*-values are for the Friedman test (non-parametric analysis of variance for dependent samples). Statistically significant differences between groups were marked by different letters (paired Wilcoxon test for dependent samples with Bonferroni correction, *p*-value < 0.05).

**Table 4:** Spearman’s correlation analysis of FSFI questionnaire domain scores for the full data.

	Desire	Arousal	Lubrication	Orgasm	Satisfaction	Pain
**desire**	1.00	0.75	0.47	0.50	0.48	0.39
**arousal**		1.00	0.65	0.75	0.69	0.64
**lubrication**			1.00	0.75	0.65	0.51
**orgasm**				1.00	0.80	0.73
**satisfaction**					1.00	0.72
**pain**						1.00

All coefficients were statistically significant (*p*-value < 0.05).

**Table 5:** Spearman’s correlation analysis of FSFI questionnaire domain scores for the women before surgery, after surgery, and follow-up.

	Desire	Arousal	Lubrication	Orgasm	Satisfaction	Pain
**desire**	1.00					
**arousal**	0.76 *^ab^	1.00			BEFORE	
**lubrication**	0.21 ^a^	0.41 *^a^	1.00			
**orgasm**	0.53 *^a^	0.80 *^ab^	0.33 *^a^	1.00		
**satisfaction**	0.08 ^a^	0.50 *^a^	0.26 ^a^	0.69 *	1.00	
**pain**	0.29	0.56 *	−0.02 ^a^	0.63 *^a^	0.70 *^ab^	1.00
**desire**	1.00					
**arousal**	0.48 *^a^	1.00			AFTER	
**lubrication**	0.49 *^a^	0.53 *^ab^	1.00			
**orgasm**	0.29 ^a^	0.47 *^a^	0.72 *^a^	1.00		
**satisfaction**	0.36 *^a^	0.48 *^a^	0.53 *^a^	0.84 *	1.00	
**pain**	0.23	0.48 *	0.56 *^b^	0.90 *^b^	0.87 *^a^	1.00
**desire**	1.00					
**arousal**	0.92 *^b^	1.00			follow-up	
**lubrication**	0.85 *^b^	0.83 *^b^	1.00			
**orgasm**	0.87 *^b^	0.89 *^b^	0.91 *^b^	1.00		
**satisfaction**	0.82 *^b^	0.82 *^b^	0.89 *^b^	0.87 *	1.00	
**pain**	0.62 *	0.72 *	0.52 *^ab^	0.59 *^a^	0.59 *^b^	1.00

Corresponding correlation coefficients for groups before, after surgery, and follow-up differing statistically significantly were marked with different letters (α = 0.05). Coefficients marked with * were statistically significant (*p*-value < 0.05).

**Table 6:** Eigenvalues, percent of cumulative explained variance, and loadings of the first three principal components (FSFI questionnaire domain scores).

	PC1	PC2	PC3
eigenvalue	4.76	0.57	0.33
cumulative variance percentage	79.35	88.87	94.43
loadings			
desire	−0.76	0.64	−0.01
arousal	−0.96	0.15	−0.04
lubrication	−0.92	−0.14	0.25
orgasm	−0.95	−0.12	0.16
satisfaction	−0.89	−0.24	0.10
pain	−0.85	−0.20	−0.48

## Data Availability

The original contributions presented in this study are included in the article. Further inquiries can be directed to the corresponding authors.

## Abbreviations

The following abbreviations are used in this manuscript:

TOT	transobturator tape
TVT	tension-free vaginal tape
UI	urinary incontinence
SUI	stress urinary incontinence
